# Editorial: Sustained Effects of Early Nutrition on Immune Development and Microbiome-Immune Crosstalk

**DOI:** 10.3389/fimmu.2020.01687

**Published:** 2020-07-31

**Authors:** Marie C. Lewis, Caroline E. Childs, Francisco J. Pérez-Cano

**Affiliations:** ^1^Food and Nutritional Sciences, School of Chemistry, Food and Pharmacy, University of Reading, Reading, United Kingdom; ^2^Faculty of Medicine, School of Human Development and Health, University of Southampton, Southampton, United Kingdom; ^3^Institute for Life Sciences, University of Southampton, Southampton, United Kingdom; ^4^Physiology Section, Department of Biochemistry and Physiology, Faculty of Pharmacy and Food Science, University of Barcelona (UB), Barcelona, Spain; ^5^Nutrition and Food Safety Research Institute (INSA.UB), Santa Coloma de Gramenet, Spain

**Keywords:** early nutrition, microbiota, immune development, developmental programming, metabolic profiles

Non-communicable diseases associated with immune system dysfunction are an increasing challenge for twenty-first century medicine. There is growing evidence that predisposition to many such conditions originate during early-life “programming events.” This term refers to critical points of developmental plasticity where changes in environmental factors have long-term effects on physiological development, including the development of the immune system.

From birth, the neonatal immune system develops rapidly in response to external stimuli, primarily from the intestinal microbiota and nutrition, and this pattern of development is essential in adult immune functionality and competence (1, 2). Nutritional components of the early diet including antioxidants, polyunsaturated fatty acids, folate, and other vitamins can directly influence immunity. This is by shifting the structure and/or functions of different immune cell populations and hematopoietic organs, and also by modulating gene expression and various signaling pathways in immune cells that contribute to homeostasis (3). Non-digestible oligosaccharides and their metabolites can also drive differential immune development indirectly through modification of both the composition and the metabolic activity of the gut microbiota (4). While current literature indicates the existence of a dynamic interplay between the immune system, nutrition and the intestinal microbiota, the molecular interactions and pathways involved in this cross-talk are complex and poorly characterized.

The adult microbiota is largely stable and even substantial interventions have limited long term impact on the composition and biochemical activity of the microbiome. However, sustained shifts in microbial ecosystems can occur in early life, especially during the neonatal period and weaning. This can be in response to nutritional factors, such as malnutrition and breastfeeding, but also in response to events such as cesarean delivery and antibiotics. Shifts in the patterns of early microbial colonization can subsequently change the development and function of the host immune system and are likely to lead to developmental imprinting. Thus, the dynamic evolution of the gut microbiota during early-life may represent a window of opportunity in which to influence the development of immunity through nutritional interventions, or the administration of specific bacterial strains (5, 6).

This Research Topic explores prenatal and postnatal dietary strategies which differentially modify the maturation of the immune system and the microbiota, and may have physiological impact later in life. Azagra-Boronat et al. investigated the effect of one of the most active and abundant human milk oligosaccharides (HMOs), 2-fucosyllactose (2-FL) in a suckling rat model. 2-FL exhibited prebiotic activity and drove the development of the microbiota to a more *Lactobacillus*-rich phenotype with increased butyrate production, and promoted the maturation of immunity in early life. In addition, 2-FL enhanced gut barrier function, possibly through the generation of divergent bacteria- and host-derived metabolic profiles.

Perdijk et al. compared the effects of another important HMO, sialyllactose (SL) with galactooligosaccharides (GOS), a prebiotic enzymatically derived from lactose that is widely used in infant formulas, using multiple *in vitro* approaches. Both prebiotic compounds induced re-epithelialization in an *in vitro* epithelial wound repair assay and induced gene expression of alkaline phosphatase, a marker of epithelial cell differentiation. However, SL and GOS differentially modified microbiota composition and SCFA production.

Taken together these studies provide further evidence that both HMOs and GOS drive important actions on the microbiota, immunity, and barrier development in early life. However, it should be noted that a single HMO cannot reflect the structure and function of the diverse oligosaccharide composition in breast milk. Breast milk provides many other important components such as cytokines, chemokines, hormones, and growth factors (7) which may also influence the microbiota and immunity and thus contribute to health in early life.

Grases-Pintó et al. assessed the role of leptin and adiponectin, which are both present in breast milk, in neonatal rats. Early supplementation with both leptin and adiponectin influenced the composition of the gut microbiota at the end of the suckling period. In addition, innate immunity-associated molecules in the intestine, including mucin, were also affected by breast milk. Their study concluded that supplementation with adipokines had an impact on the phenotype of those immune cells in close contact with the intestinal milieu, the intraepithelial lymphocytes. This reinforces the idea that nutritional programming of the immune system by breast milk may be driven by factors other than oligosaccharides.

One of the most prevalent food allergies during the first year of life is to cow's milk proteins (CMP) and the incidence is increasing rapidly. Guadamuro et al. demonstrated that the introduction of intact CMP to infants with non-IgE mediated CMP allergy changed both immune mediators and the composition and metabolic end-products of the microbiota. The authors also identified that consumption of the probiotic *L. rhamnosus* GG during CMP allergy may have contributed to gut homeostasis by fine-tuning these profiles.

Sexual dimorphism can also influence immunity in an out bred infant pig model as identified by Christoforidou et al.. Early sex disparities were found to underlie both mucosal immune development and systemic responses to novel antigen. Crucially, significant effects on immune development of nutritional interventions with inulin or *Bifidobacterium lactis* were also sex dependent. The importance of sex-stratifying nutritional interventions, even in very young animals, could translate to personalized recommendations for human infants.

Together, these results demonstrate that early nutrition can significantly modify the gut environment in infants and have positive impacts on immune, metabolic, and microbiota development during early life. Since the development of these systems is an important determining factor in later health, it follows that early nutrition could have a lasting impact on lifecourse health. This Research Topic therefore provides new insights to support the development of therapeutic dietary interventions to achieve optimal immune and metabolic health.

## Author Contributions

All authors listed have made a substantial, direct and intellectual contribution to the work, and approved it for publication.

## Conflict of Interest

The authors declare that the research was conducted in the absence of any commercial or financial relationships that could be construed as a potential conflict of interest.
